# Mental health and initiation of antiretroviral treatment at enrolment into HIV care in Cameroon under a national “treat all” policy: a cross‐sectional analysis

**DOI:** 10.1002/jia2.25842

**Published:** 2021-11-22

**Authors:** Angela M. Parcesepe, Lindsey M. Filiatreau, Peter Vanes Ebasone, Anastase Dzudie, Brian W. Pence, Milton Wainberg, Marcel Yotebieng, Kathryn Anastos, Eric Pefura‐Yone, Rogers Ajeh, Denis Nash

**Affiliations:** ^1^ Department of Maternal and Child Health Gillings School of Global Public Health University of North Carolina at Chapel Hill Chapel Hill North Carolina USA; ^2^ University of North Carolina at Chapel Hill Carolina Population Center Chapel Hill North Carolina USA; ^3^ Gillings School of Global Public Health Department of Epidemiology University of North Carolina at Chapel Hill Chapel Hill North Carolina USA; ^4^ Clinical Research Education Networking and Consultancy Yaounde Cameroon; ^5^ Department of Psychiatry New York State Psychiatric Institute and Columbia University New York New York USA; ^6^ Department of Medicine Albert Einstein College of Medicine Bronx New York USA; ^7^ Departments of Medicine and Epidemiology & Population Health Albert Einstein College of Medicine Bronx New York USA; ^8^ Jamot Hospital Yaounde Cameroon; ^9^ City University of New York Institute of Implementation Science in Population Health Graduate School of Public Health and Health Policy New York New York USA

**Keywords:** Africa, anxiety, ART initiation, Cameroon, depression, mental health

## Abstract

**Introduction:**

Rapid antiretroviral treatment (ART) initiation reduces time from HIV infection to viral suppression, decreasing HIV transmission risk. Mental health symptoms may influence timing of ART initiation. This study estimated the prevalence of ART initiation at enrolment into HIV care and the relationship between mental health and ART initiation at enrolment into HIV care.

**Methods:**

We conducted interviews with 426 individuals initiating HIV care in Cameroon between June 2019 and March 2020 to estimate the association between mental health and timing of ART initiation. Depression (Patient Health Questionnaire‐9; cut‐point 10), anxiety (Generalized Anxiety Disorder‐7; cut‐point 10), post‐traumatic stress disorder (PTSD) (PTSD Checklist for DSM‐5; cut‐point 31) and harmful alcohol use (Alcohol Use Disorders Identification Test; cut‐point 16) were dichotomized to represent those with and without each exposure at first HIV care appointment. Date of ART initiation (date ART prescribed) was ascertained from medical records. Separate multivariable log‐binomial regression models were used to estimate the association between mental health exposures and ART initiation at enrolment into care.

**Results and discussion:**

Overall, 87% initiated ART at enrolment into HIV care. Approximately 20% reported depressive symptoms, 15% reported PTSD symptoms, 12% reported anxiety symptoms and 13% reported harmful alcohol use. In multivariable analyses, individuals with moderate to severe depressive symptoms had 1.7 (95% confidence interval [CI] 1.1, 2.7) times the prevalence of not initiating ART at enrolment into HIV care compared to those with no or mild depressive symptoms. Those with symptoms of PTSD, compared to those without, had 1.9 (95% CI 1.2, 2.9) times the prevalence of not initiating ART at enrolment into HIV care. Symptoms of anxiety or harmful drinking were not associated with ART initiation at enrolment into HIV care in multivariable models.

**Conclusions:**

Symptoms of depression and PTSD were associated with lower prevalence of ART initiation at enrolment into HIV care among this sample of individuals initiating HIV care in Cameroon under a “treat all” policy. Research should examine barriers to timely ART initiation, whether incorporating mental health services into HIV care improves timely ART initiation, and whether untreated symptoms of depression and PTSD drive suboptimal HIV care outcomes.

## 1 INTRODUCTION

The World Health Organization strongly recommends initiation of antiretroviral treatment (ART) as soon as possible after HIV diagnosis [1]. Rapid ART initiation has been associated with improved linkage to and retention in care and reduced time from HIV infection to viral suppression, thereby lowering the risk of HIV transmission, though evidence is mixed across sub‐Saharan Africa (SSA) [2, 3, 4].

“Treat all” policies, in which all people living with HIV (PLWH) are eligible to initiate ART as soon as possible after diagnosis, have been widely implemented throughout SSA. Despite such policies, delays in ART initiation persist [5, 6, 7]. Cameroon began implementation of a national “treat all” policy in 2016. Approximately 500,000 people are living with HIV throughout Cameroon, representing 3% of adults [8]. Cameroon remains far from achieving the UNAIDS 95‐95‐95 goals, with 74% of PLWH on ART [8].

Little is known about factors associated with rapid ART initiation throughout SSA, including in Cameroon. Mental health disorders are among the most common comorbidities among PLWH and have been associated with suboptimal outcomes throughout the HIV care cascade, including delayed ART initiation [9, 10, 11]. However, the extent to which mental health symptoms are associated with rapid ART initiation in the context of treat all implementation in resource‐limited settings remains largely unexamined. Greater understanding of the relationship between mental health and the timing of ART initiation can inform strategies to increase more timely ART initiation and has the potential to improve the health and well‐being of PLWH with co‐morbid mental health disorders.

This study aimed to estimate the: (1) prevalence of ART initiation at enrolment into HIV care and (2) relationship between mental health symptoms and ART initiation at enrolment into HIV care among a cohort of PLWH in Cameroon.

## 2 METHODS

### 2.1 Data collection

Data were collected from in‐person interviews with 426 individuals initiating HIV care at three urban HIV treatment clinics in Cameroon between June 2019 and March 2020. Study sites were selected because they were participating in the Central Africa International epidemiology Databases to Evaluate AIDS (CA‐IeDEA) Cameroon study. IeDEA is a research consortium established to collect and harmonize global HIV data [12]. Individuals were eligible to participate if they were 21 years or older and newly enrolling in HIV care at one of the three HIV clinics. Data collection consisted of one structured interview, which included questions on mental health and substance use and was conducted by a trained research assistant. Study participants also had their CD4 cell counts measured at the HIV care facility within 2 weeks of enrolment into HIV care. This study was approved by the Institutional Review Board at the University of North Carolina at Chapel Hill and the National Ethical Committee of Research for Human Health in Cameroon. All participants provided written informed consent.

### 2.2 Measures

#### 2.2.1 ART initiation at enrolment into HIV care

Individuals who initiated ART on the same day they enrolled in HIV care were categorized as having initiated ART at enrolment into HIV care. Individuals who initiated ART one or more days after enrolment into HIV care were categorized as not having initiated ART at enrolment in HIV care.

#### 2.2.2 Depressive symptoms

Depressive symptoms were assessed with the Patient Health Questionnaire‐9 (PHQ‐9), a 9‐item screener that assesses the presence of depressive symptoms within the last 2 weeks [13]. Scores of 10 or greater are commonly considered an indication of moderate to severe depressive symptoms [13]. The PHQ‐9 has been previously validated with PLWH in SSA [14, 15, 16].

#### 2.2.3 Anxiety symptoms

Anxiety symptoms were assessed with the Generalized Anxiety Disorder‐7 (GAD‐7), a 7‐item screener that assesses the presence of anxiety symptoms within the past 2 weeks [17]. Scores of 10 or greater are commonly considered an indication of moderate or severe anxiety symptoms. The GAD‐7 has been validated in clinical and research environments across cultural settings, including among a primary care population with a high prevalence of HIV in SSA [18, 19, 20].

#### 2.2.4 Post‐traumatic stress disorder symptoms

Post‐traumatic stress disorder (PTSD) symptoms were assessed with the PTSD Checklist for DSM‐5 (PCL‐5), a 20‐item screener that assesses the presence of PTSD symptoms in the past month [21]. Scores of 31 or greater are indicative of probable PTSD [22]. The PCL‐5 has been validated across a range of cultural settings, including among a primary care population with a high prevalence of HIV in Zimbabwe [23, 24, 25].

#### 2.2.5 Alcohol use

Alcohol use was measured using the 10‐item Alcohol Use Disorders Identification Test (AUDIT) [26]. Scores of 16 or greater were considered indicative of harmful drinking or potential alcohol use disorder. This scale has been validated and used in populations with high HIV prevalence in SSA [27, 28].

#### 2.2.6 Symptoms of depression, anxiety or PTSD

We created a dichotomous variable to represent individuals with and without symptoms of depression, anxiety or PTSD (0 = screened negative for symptoms of depression, anxiety and PTSD; 1 = screened positive for symptoms of depression, anxiety or PTSD).

#### 2.2.7 Symptoms of depression, anxiety, PTSD or harmful alcohol use

We created a dichotomous variable to represent individuals with and without symptoms of depression, anxiety, PTSD or harmful alcohol use (0 = screened negative for symptoms of depression, anxiety, PTSD and harmful alcohol use; 1 = screened positive for symptoms of depression, anxiety, PTSD or harmful alcohol use).

Socio‐demographic characteristics explored included age, gender, education, relationship status, employment, time away from home and number of children.

#### 2.2.8 Missing CD4 data

Approximately midway through data collection (November 2019), CD4 testing ceased being available at two of the health facilities at which this survey was conducted. As a result, CD4 cell count was missing for 180 (42%) study participants.

### 2.3 Data analysis

Univariate analyses were conducted to assess the prevalence of ART initiation at enrolment into HIV care. Bivariate analyses between each mental health exposure and ART initiation at enrolment into HIV care were conducted using Pearson chi‐squared tests. Wilcoxon rank‐sum tests were conducted to compare median CD4 cell count at engagement in HIV care among those with and without ART initiation at enrolment into HIV care. Separate multivariable log‐binomial regression models were used to estimate the prevalence ratio of each mental health exposure with ART initiation at enrolment into HIV care as well the association between any mental health exposure assessed and ART initiation at enrolment into HIV care. All adjusted analyses controlled a priori for age, gender and health facility [29].

## 3 RESULTS AND DISCUSSION

A total of 426 individuals enrolled in the study. Timing of ART initiation was available for 420 participants. Among 420 included participants, 59% (*n* = 246) were women (Table 1). Overall, 87% (*n* = 365) of participants initiated ART at enrolment into HIV care. It is worth noting that among those who delayed ART initiation, most (75%) subsequently initiated ART within 1 week of HIV care engagement and, thus, may not have experienced a clinically meaningful delay in ART initiation. Among individuals for whom CD4 data were available, median CD4 cell count did not differ meaningfully between those with and without ART initiation at enrolment into HIV care. Approximately 20% (*n* = 83) of participants reported symptoms of depression, 15% (*n* = 63) reported symptoms of PTSD, 12% (*n* = 49) reported symptoms of anxiety and 13% (*n* = 56) of participants reported symptoms of harmful alcohol use (Table 2).

**Table 1:** Characteristics of 420 people living with HIV newly initiating HIV care in Cameroon by ART initiation at enrolment into HIV care

		ART initiation at enrolment into HIV care		
	Total sample *n* = 420*N* (%)	Yes, *n* = 365*N* (%)	No, *n* = 55*N* (%)	*p*‐value
Gender				
Men	174 (41.4)	150 (41.1)	24 (43.6)	0.72
Women	246 (58.6)	215 (58.9)	31 (56.4)	
Age in years				
21–39	246 (58.6)	218 (59.7)	28 (50.9)	0.22
40+	174 (41.4)	147 (40.3)	27 (49.1)	
Education				
None	30 (7.1)	26 (7.1)	4 (7.3)	0.66
Primary	214 (51.0)	183 (50.1)	31 (56.4)	
Secondary	176 (41.9)	156 (42.7)	20 (36.4)	
Religion				
Catholic	155 (36.9)	142 (38.9)	13 (23.6)	0.07
Protestant	137 (32.6)	119 (32.6)	18 (32.7)	
Born again Christian/Evangelical	90 (21.4)	72 (19.7)	18 (32.7)	
Other	38 (9.0)	32 (8.8)	6 (10.9)	
Relationship status				
Single	175 (41.7)	154 (42.2)	21 (38.2)	0.57
Partnered	245 (58.3)	211 (57.8)	34 (61.8)	
Number of children^a^				
0	79 (18.9)	67 (18.4)	12 (22.2)	0.50
1+	339 (81.1)	297 (81.6)	42 (77.8)	
Away from home >1 month in past year				
Yes	163 (38.8)	134 (36.7)	29 (52.7)	0.02
No	257 (61.2)	231 (63.3)	26 (47.3)	
Employment status				
Working for pay	271 (64.5)	240 (65.8)	31 (56.4)	0.17
Not working for pay	149 (35.5)	125 (34.2)	24 (43.6)	
Median days to ART initiation, (IQR), [range]	0, (0–0), [0–34]	0 (0–0), [0–0]	2,(1–7), [1–34]	<0.0001
Median CD4 at engagement in HIV care (IQR), cells/μL^a^	192 (99–322)	192 (93–326)	190.5 (120–315)	0.93

^a^ Missing: Number of children *n* = 2; CD4 at engagement in HIV care *n* = 180.

Abbreviations: ART, antiretroviral treatment; HIV, human immunodeficiency virus; IQR, interquartile range.

**Table 2:** Mental health symptoms and ART initiation at enrolment into HIV care among 420 people living with HIV newly initiating HIV care in Cameroon

		ART initiation at enrolment into HIV care			
	Total sample *n* = 420*N* (%)	Yes, *n* = 365*N* (%)	No, *n* = 55*N* (%)	Bivariate PR (95% CI)	Multivariable^e^ aPR (95% CI)
Depression^a^					
No/mild symptoms	331 (80.0)	299 (83.1)	32 (59.3)	REF	REF
Moderate/severe symptoms	83 (20.0)	61 (16.9)	22 (40.7)	2.74 (1.68, 4.46)	1.68 (1.05, 2.67)
PTSD^b^					
No probable PTSD	344 (84.5)	312 (88.1)	32 (60.4)	REF	REF
Probable PTSD	63 (15.5)	42 (11.9)	21 (39.6)	3.58 (2.22, 5.79)	1.86 (1.18, 2.92)
Anxiety^c^					
No/mild symptoms	359 (88.0)	316 (89.3)	43 (79.6)	REF	REF
Moderate/severe symptoms	49 (12.0)	38 (10.7)	11 (20.4)	1.87 (1.04, 3.38)	1.34 (0.75, 2.38)
Harmful alcohol use^d^					
No	362 (86.6)	315 (86.8)	47 (85.4)	REF	REF
Yes	56 (13.4)	48 (13.2)	8 (14.6)	1.10 (0.55, 2.20)	0.86 (0.45, 1.63)
Symptoms of depression, PTSD or anxiety					
No	280 (70.7)	256 (74.8)	24 (44.4)	REF	REF
Yes	116 (29.3)	86 (25.2)	30 (55.6)	3.02 (1.85, 4.93)	1.91 (1.17, 3.12)
Symptoms of depression, PTSD, anxiety or harmful alcohol use					
No	242 (61.1)	222 (64.9)	20 (37.0)	REF	REF
Yes	154 (38.9)	120 (35.1)	34 (63.0)	2.67 (1.60, 4.47)	1.69 (1.04, 2.74)

Note: Missing: depression *n* = 6; PTSD *n* = 13; anxiety *n* = 12; harmful alcohol use *n* = 2; Depression, PTSD or anxiety *n* = 24; depression, PTSD, anxiety or harmful alcohol use *n* = 24.

^a^ Models include depression, but not anxiety, PTSD or harmful alcohol use.

^b^ Models include PTSD, but not depression, anxiety or harmful alcohol use.

^c^ Models include anxiety, but not depression, PTSD or harmful alcohol use.

^d^ Models include harmful alcohol use, but not depression, anxiety or PTSD.

^e^ Models adjusted for age, gender and health facility.

Symptoms of depression and PTSD were associated with delayed ART initiation (i.e. not the same day as enrolment into HIV care). Specifically, in multivariable analyses, individuals with moderate to severe depressive symptoms had 1.7 (95% confidence interval [CI] 1.1, 2.7) times the prevalence of delayed ART initiation compared to those with no or mild depressive symptoms (Table 2). Similarly, individuals with symptoms of PTSD had 1.9 (95% CI 1.2, 2.9) times the prevalence of delayed ART initiation compared to those without symptoms of PTSD. Taken together, individuals with symptoms of depression, anxiety or PTSD had 1.9 (95% CI 1.2, 3.1) times the prevalence of delayed ART initiation compared to those without symptoms of any of these disorders. Similarly, individuals with symptoms of depression, anxiety, PTSD or harmful alcohol use had 1.7 (95% CI 1.0, 2.7) times the prevalence of delayed ART initiation compared to those without symptoms of any of these disorders. In multivariable analyses, symptoms of harmful alcohol use and symptoms of moderate or severe anxiety were not meaningfully associated with timing of ART initiation (harmful alcohol use: adjusted prevalence ratio [aPR] 0.9 (95% CI 0.4, 1.6); anxiety: aPR 1.3 (95% CI 0.7, 2.4)).

The mechanisms through which symptoms of depression and PTSD may influence timing of ART initiation are unclear. In particular, the extent to which delayed ART initiation among individuals with depression or PTSD symptoms in this sample was a result of patient‐ versus provider‐level decisions, or both, is unknown. For example, it is possible that delayed ART initiation among patients with depression or PTSD was related to lower levels of patient readiness to initiate ART among PLWH with these symptoms. It is also possible that lower prevalence of ART initiation at enrolment into HIV care among PLWH with symptoms of depression or PTSD was influenced by providers’ perceptions of patient readiness or concerns about patients’ physical or mental health. Future research should examine system‐, provider‐ and patient‐level barriers to ART initiation at enrolment into HIV care. The extent to which incorporating mental health services into HIV care may facilitate more timely ART initiation should be investigated.

Symptoms of anxiety and harmful alcohol use were not associated with ART initiation at enrolment into HIV care in multivariable analyses. While research remains limited, our findings are similar to those with PLWH in Ethiopia that found that alcohol use was not associated with same‐day ART initiation in multivariable models [7]. It is possible that cultural norms related to alcohol use influence providers’ decisions to screen for or address alcohol use among patients and providers’ decisions about initiating ART among PLWH who report harmful alcohol use. Future research examining the relationship between cultural norms, alcohol use and ART initiation is warranted. The authors are not aware of previous research that examined the relationship between anxiety and ART initiation at enrolment into HIV care. However, one study with men who have sex with men in China found that anxiety was associated with earlier ART initiation [29]. Anxiety disorders are generally less likely to be recognized and treated compared to depression and PTSD [30]. The relationship between anxiety and timing of ART initiation may be influenced by limited recognition and diagnosis of anxiety disorders among providers.

Potential mediating or modifying factors, such as self‐efficacy, that influence the relationship between symptoms of depression or PTSD and ART initiation should be examined. Much research on timing of ART initiation has focused on the relationship between socio‐demographic factors and ART initiation. For example, among PLWH in South Africa, women were more likely than men to initiate ART on the same day as diagnosis, while older individuals were less likely than younger adults to initiate ART on the same day as diagnosis [31]. However, neither age nor gender was associated with the prevalence of ART initiation at enrolment into HIV care in the current study.

This study has limitations worth noting. All data were collected at enrolment into HIV care. Because date of first HIV‐positive diagnosis was not available, the current analysis could not examine the relationship between timing of HIV diagnosis and ART initiation at enrolment into HIV care. The extent to which ART initiation at enrolment into HIV care differs between those who are and are not newly diagnosed warrants further investigation. Further, data were collected from three urban hospital‐based HIV treatment clinics in Cameroon and may not be generalizable to other populations or settings.

## 4 CONCLUSIONS

Although ART initiation at enrolment into HIV care was common among this sample of PLWH in Cameroon, approximately one in seven individuals experienced delayed ART initiation. Individuals with symptoms of depression and PTSD at entry into HIV care were more likely to experience loss to care and/or delayed ART initiation. Greater understanding of mechanisms through which depression and PTSD may influence timing of ART initiation is warranted. Research into multilevel barriers to rapid ART initiation and the extent to which these barriers influence the relationship between mental health and ART initiation at enrolment into HIV care is needed.

## COMPETING INTEREST

The authors have no competing interest to report.

## AUTHORS' CONTRIBUTIONS

AMP: conceptualization, funding, analysis, writing – original draft; LMF: analysis, writing – review and editing; PVE and EPY: project administration, writing – review and editing; AD: project administration, supervision, writing – review and editing; RA, MY, and KA: writing – review and editing; MW, BP, and DN: conceptualization, writing – review and editing. All authors have read and approved the final manuscript.

## FUNDING

This research was supported by NIMH grant K01 MH114721 and NICHD grant P2C HD050924 (Carolina Population Center), NIAID grant P30 AI050410 (UNC Center for AIDS Research) and NIAID grant U01AI096299.

## Data Availability

The dataset contains sensitive information and is not publicly available. However, it could be made available from the first author (AMP) on reasonable request, with approval from the IRB at the University of North Carolina at Chapel Hill to maintain confidentiality.

## References

[1] World Health Organization . Guidelines for managing advanced HIV disesase and rapid initiation of antiretroviral therapy. Geneva: World Health Organization; 2017. 29341560

[2] Cohen MS , Chen YQ , McCauley M , Gamble T , Hosseinipour MC , Kumarasamy N , et al. Prevention of HIV‐1 infection with early antiretroviral therapy. N Engl J Med. 2011; 365(6):493–505. 21767103 10.1056/NEJMoa1105243 PMC3200068

[3] Cohen MS , Chen YQ , McCauley M , Gamble T , Hosseinipour MC , Kumarasamy N , et al. Antiretroviral therapy for the prevention of HIV‐1 transmission. N Engl J Med. 2016; 375(9):830–9. 27424812 10.1056/NEJMoa1600693 PMC5049503

[4] Pilcher CD , Ospina‐Norvell C , Dasgupta A , Jones D , Hartogensis W , Torres S , et al. The effect of same‐day observed initiation of antiretroviral therapy on HIV viral load and treatment outcomes in a US public health setting. J Acquir Immune Defic Syndr. 2017; 74(1):44–51. 27434707 10.1097/QAI.0000000000001134 PMC5140684

[5] Tymejczyk O , Brazier E , Yiannoutsos CT , Vinikoor M , van Lettow M , Nalugoda F , et al. Changes in rapid HIV treatment initiation after national “treat all” policy adoption in 6 sub‐Saharan African countries: regression discontinuity analysis. PLoS Med. 2019; 16(6):e1002822. 31181056 10.1371/journal.pmed.1002822 PMC6557472

[6] Onoya D , Sineke T , Hendrickson C , Mokhele I , Maskew M , Long LC , et al. Impact of the test and treat policy on delays in antiretroviral therapy initiation among adult HIV positive patients from six clinics in Johannesburg, South Africa: results from a prospective cohort study. BMJ Open. 2020; 10(3):e030228. 10.1136/bmjopen-2019-030228 PMC7170559 32213514

[7] Moges NA , Adesina OA , Okunlola MA , Berhane Y . Same‐day antiretroviral treatment (ART) initiation and associated factors among HIV positive people in Northwest Ethiopia: baseline characteristics of prospective cohort. Arch Public Health. 2020; 78:87. 32983450 10.1186/s13690-020-00473-4 PMC7510057

[8] UNAIDS . Country overview: Cameroon. 2020.

[9] Truong M , Rane MS , Govere S , Galagan SR , Moosa MY , Stoep AV , et al. Depression and anxiety as barriers to art initiation, retention in care, and treatment outcomes in KwaZulu‐Natal, South Africa. EClinicalMedicine. 2021; 31:100621. 33490927 10.1016/j.eclinm.2020.100621 PMC7806795

[10] Levintow SN , Pence BW , Powers KA , Breskin A , Sripaipan T , Ha TV , et al. Depression, antiretroviral therapy initiation, and HIV viral suppression among people who inject drugs in Vietnam. J Affect Disord. 2021; 281:208–15. 33333474 10.1016/j.jad.2020.12.024 PMC7855445

[11] Tegger MK , Crane HM , Tapia KA , Uldall KK , Holte SE , Kitahata MM . The effect of mental illness, substance use, and treatment for depression on the initiation of highly active antiretroviral therapy among HIV‐infected individuals. AIDS Patient Care STDs. 2008; 22(3):233–43. 18290749 10.1089/apc.2007.0092

[12] Chammartin F , Dao Ostinelli CH , Anastos K , Jaquet A , Brazier E , Brown S , et al. International epidemiology databases to evaluate AIDS (IeDEA) in sub‐Saharan Africa, 2012–2019. BMJ Open. 2020; 10(5):e035246. 10.1136/bmjopen-2019-035246 PMC7232622 32414825

[13] Kroenke K , Spitzer RL , Williams JB . The PHQ‐9: validity of a brief depression severity measure. J Gen Intern Med. 2001; 16(9):606–13. 11556941 10.1046/j.1525-1497.2001.016009606.x PMC1495268

[14] Pence BW , Gaynes BN , Atashili J , O'Donnell JK , Tayong G , Kats D , et al. Validity of an interviewer‐administered patient health questionnaire‐9 to screen for depression in HIV‐infected patients in Cameroon. J Affect Disord. 2012; 143(1–3):208–13. 22840467 10.1016/j.jad.2012.05.056 PMC3500577

[15] Monahan PO , Shacham E , Reece M , Kroenke K , Ong'or WO , Omollo O , et al. Validity/reliability of PHQ‐9 and PHQ‐2 depression scales among adults living with HIV/AIDS in western Kenya. J Gen Intern Med. 2009; 24(2):189–97. 19031037 10.1007/s11606-008-0846-z PMC2629000

[16] Akena D , Joska J , Obuku EA , Stein DJ . Sensitivity and specificity of clinician administered screening instruments in detecting depression among HIV‐positive individuals in Uganda. AIDS Care. 2013; 25(10):1245–52. 23398282 10.1080/09540121.2013.764385

[17] Spitzer RL , Kroenke K , Williams JB , Lowe B . A brief measure for assessing generalized anxiety disorder: the GAD‐7. Arch Intern Med. 2006; 166(10):1092–7. 16717171 10.1001/archinte.166.10.1092

[18] Chibanda D , Verhey R , Gibson LJ , Munetsi E , Machando D , Rusakaniko S , et al. Validation of screening tools for depression and anxiety disorders in a primary care population with high HIV prevalence in Zimbabwe. J Affect Disord. 2016; 198:50–5. 27011359 10.1016/j.jad.2016.03.006

[19] Zhong QY , Gelaye B , Zaslavsky AM , Fann JR , Rondon MB , Sánchez SE , et al. Diagnostic validity of the Generalized Anxiety Disorder ‐ 7 (GAD‐7) among pregnant women. PLoS One. 2015; 10(4):e0125096. 25915929 10.1371/journal.pone.0125096 PMC4411061

[20] Ruiz MA , Zamorano E , García‐Campayo J , Pardo A , Freire O , Rejas J . Validity of the GAD‐7 scale as an outcome measure of disability in patients with generalized anxiety disorders in primary care. J Affect Disord. 2011; 128(3):277–86. 20692043 10.1016/j.jad.2010.07.010

[21] Blevins CA , Weathers FW , Davis MT , Witte TK , Domino JL . The Posttraumatic Stress Disorder Checklist for DSM‐5 (PCL‐5): development and initial psychometric evaluation. J Trauma Stress. 2015; 28(6):489–98. 26606250 10.1002/jts.22059

[22] Geier TJ , Hunt JC , Nelson LD , Brasel KJ , deRoon‐Cassini TA . Detecting PTSD in a traumatically injured population: the diagnostic utility of the PTSD Checklist for DSM‐5. Depress Anxiety. 2019; 36(2):170–8. 30597679 10.1002/da.22873 PMC6373876

[23] Verhey R , Chibanda D , Gibson L , Brakarsh J , Seedat S . Validation of the posttraumatic stress disorder checklist‐5 (PCL‐5) in a primary care population with high HIV prevalence in Zimbabwe. BMC Psychiatry. 2018; 18(1):109. 29685117 10.1186/s12888-018-1688-9 PMC5913864

[24] Ibrahim H , Ertl V , Catani C , Ismail AA , Neuner F . The validity of Posttraumatic Stress Disorder Checklist for DSM‐5 (PCL‐5) as screening instrument with Kurdish and Arab displaced populations living in the Kurdistan region of Iraq. BMC Psychiatry. 2018; 18(1):259. 30115040 10.1186/s12888-018-1839-z PMC6097219

[25] Ashbaugh AR , Houle‐Johnson S , Herbert C , El‐Hage W , Brunet A . Psychometric validation of the English and French versions of the Posttraumatic Stress Disorder Checklist for DSM‐5 (PCL‐5). PLoS One. 2016; 11(10):e0161645. 27723815 10.1371/journal.pone.0161645 PMC5056703

[26] Saunders JB , Aasland OG , Babor TF , de la Fuente JR , Grant M . Development of the Alcohol Use Disorders Identification Test (AUDIT): WHO Collaborative Project on Early Detection of Persons with Harmful Alcohol Consumption–II. Addiction. 1993; 88(6):791–804. 8329970 10.1111/j.1360-0443.1993.tb02093.x

[27] Adewuya AO . Validation of the alcohol use disorders identification test (audit) as a screening tool for alcohol‐related problems among Nigerian university students. Alcohol Alcohol. 2005; 40(6):575–7. 16115823 10.1093/alcalc/agh197

[28] Blair AH , Pearce ME , Katamba A , Malamba SS , Muyinda H , Schechter MT , et al. The Alcohol Use Disorders Identification Test (AUDIT): exploring the factor structure and cutoff thresholds in a representative post‐conflict population in Northern Uganda. Alcohol Alcohol. 2017; 52(3):318–27. 28003244 10.1093/alcalc/agw090

[29] Tao J , Vermund SH , Lu H , Ruan Y , Shepherd BE , Kipp AM , et al. Impact of depression and anxiety on initiation of antiretroviral therapy among men who have sex with men with newly diagnosed HIV infections in China. AIDS Patient Care STDs. 2017; 31(2):96–104. 28170305 10.1089/apc.2016.0214 PMC5312604

[30] DeMartini J , Patel G , Fancher TL . Generalized anxiety disorder. Ann Intern Med. 2019;170(7):Itc49–64. 30934083 10.7326/AITC201904020

[31] Lilian RR , Rees K , McIntyre JA , et al. Same‐day antiretroviral therapy initiation for HIV‐infected adults in South Africa: Analysis of routine data. PloS One 2020;15(1):e0227572. 31935240 10.1371/journal.pone.0227572 PMC6959580

